# Use of Dermal Regeneration Template in Closure of a Large Vesicovaginal Fistula: An Initial Report

**DOI:** 10.1055/s-0045-1801867

**Published:** 2025-01-21

**Authors:** Guruswamy Vishwanath, Ankur Modi

**Affiliations:** 1 Department Plastic and Reconstructive Surgery, Dr. D. Y. Patil Medical College, Hospital and Research Center, Dr. D. Y. Patil Vidyapeeth, Pune, Maharashtra, India

Sir,

The value of dermal regeneration templates (DRTs) is well established in skin grafting. Newer uses for DRT are emerging. We report a case in which DRT was used in successful closure of a large vesicovaginal fistula (VVF).

**Fig. 1:**
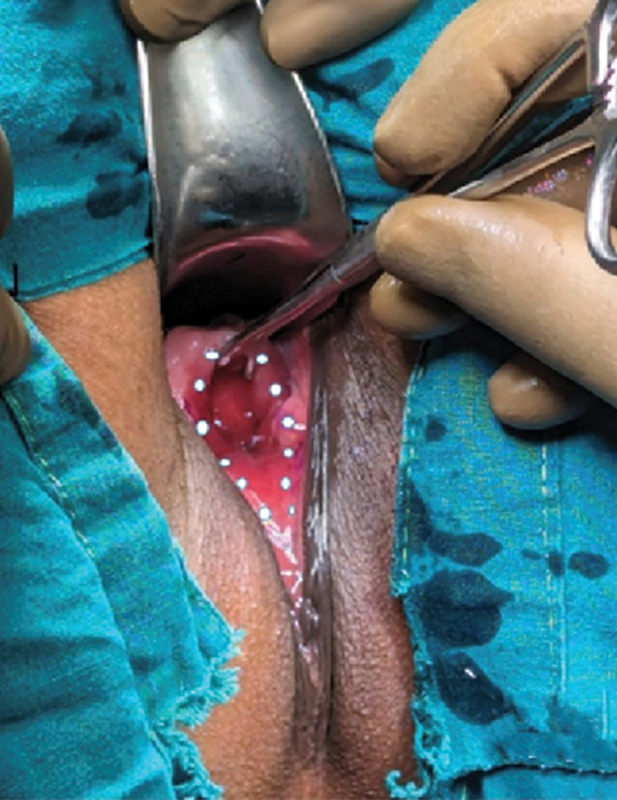
Extent of fistula is shown, outlined by white dots. Fistula involves the posterior wall and trigone of the bladder, bladder neck, and urethra including the external urethral meatus.

A primipara presented with incontinence of a year's duration following caesarian section. Physical examination showed a single very large VVF involving the posterior wall and trigone of the bladder, bladder neck, and urethra including the external urethral meatus ( Fig. 1 ). All radiological and hematological investigations were normal.

**Fig. 2:**
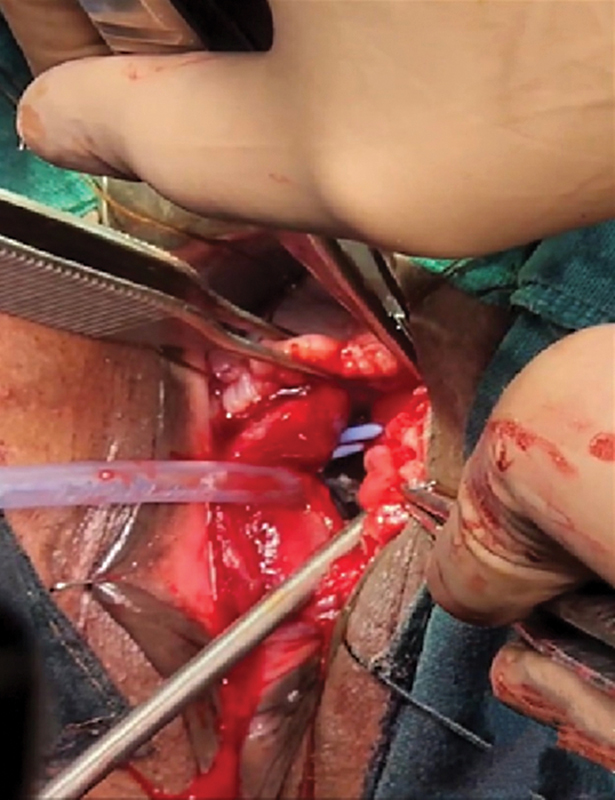
Margins of the fistula are being dissected to separate bladder and urethra from vagina. Ureteric catheters (blue) have been placed to protect the ureters during dissection, and can be seen through the vagina.

**Fig. 3:**
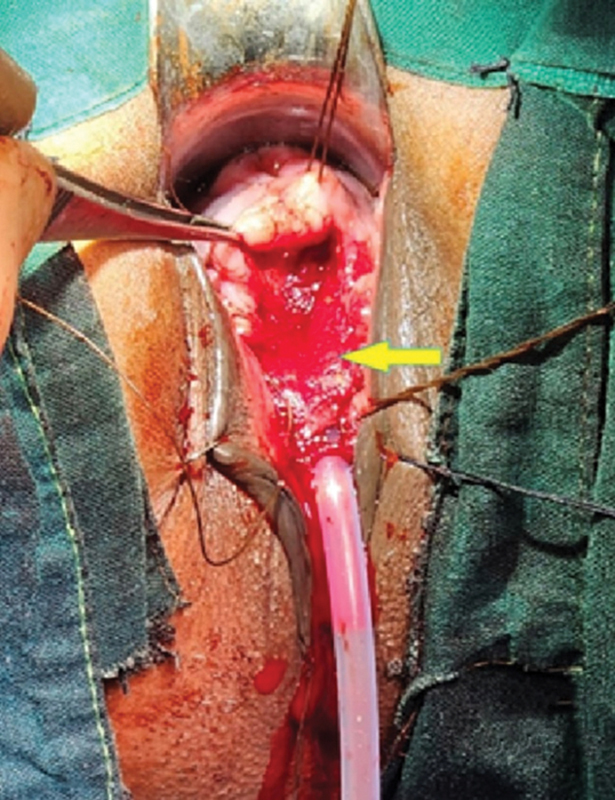
Dermal regeneration template (Matriderm) (indicated by solid yellow arrow) has been placed over the bladder and urethral closure for interposition prior to closure of the vaginal wall.

The patient was taken up for surgery. Ureteric catheters were placed through cystoscopy, and the patient then placed in the jackknife position. The margins of the fistula were incised, the bladder and urethra separated from the vagina ( Fig. 2 ), and closed over a Foley's catheter, with special attention to closure of the bladder neck region. DRT (Matriderm, 1-mm thickness, fenestrated, 52 × 37 mm) was then placed over the suture line to cover it ( Fig. 3 ). The margins of the vaginal mucosa were thereafter approximated over the DRT, completing the repair. A vaginal pack was placed and removed on the 5th postoperative day.

**Fig. 4:**
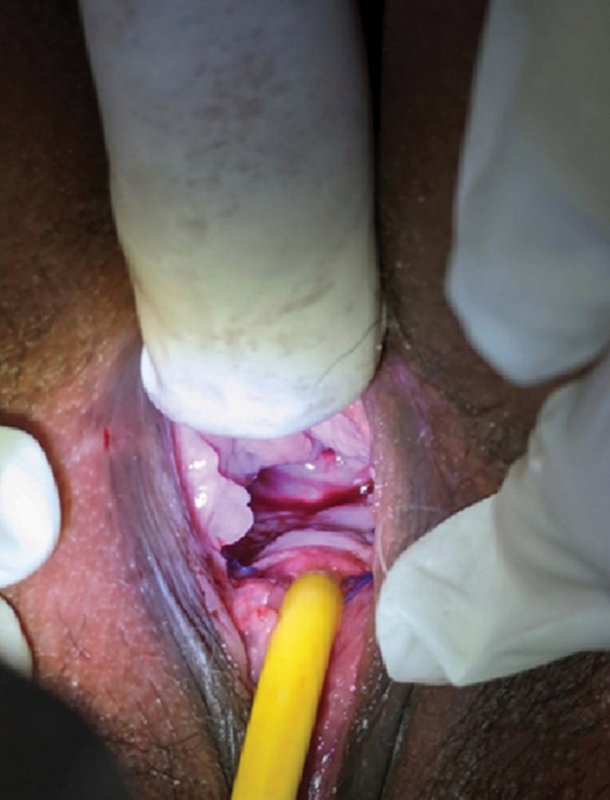
Appearance at 4 weeks postoperatively. Healing is good, complete separation of the urinary and genital tracts is seen, with no recurrence of fistula.

The urethral catheter was removed at 4 weeks. Physical examination showed excellent healing, complete separation of urinary and genital tracts without recurrence of fistula with good continence ( Fig. 4 ). At 6 months of follow up, the patient has no recurrence.

VVFs are vexing problems. Operating large, multiple, or recurrent VVFs in a scarred environment can be challenging; reported success rates vary between 53.8 and 100%. 1

Tension free closure with non-overlapping suture lines, improved vascularity, obliteration of dead space, continuous postoperative drainage of the bladder and physical interposition of tissue in the repair improve success rates. 2

DRT is acknowledged for its rapid vascularization and integration in the body, and undergoes transformation to connective tissue promoting sound healing. DRT when interposed between repaired viscera would undergo rapid vascularization and integration from both sides of the template, promoting healing and providing effective interposition.

DRT has been used successfully for interposition in several situations such as between implants and nerves to protect the latter, 3 in hemifacial atrophy to provide stable bulk, 4 in the management of palatal 5 and urethral fistulae, and even to treat exposure of the brain.

The use of DRT in VVF closure and similar situations merits further study.
